# Wood’s Medical and Surgical Monographs

**Published:** 1889-03

**Authors:** 


**Wood's Medical and Surgical Monographs.** Vol. I., No. 2. February, 1889. Contents: 1. Gonorrheal Infection in Women. By William Jap Sinclair, M. A., M. D.; 2. On Giddiness. By Thomas Grainger Stewart, M. D.; 3. Albuminuria in Bright's Diseases. By Dr. Pierre Jaenton, Paris, France. 8vo, pp. 260. New York: Wm. Wood & Co., 56 and 58 Lafayette Place.

This second number of this new series of medical publications impresses us even more favorably, if it were possible, than the first, an extended notice of which appeared in the January number of this journal.

The first subject in the number now under consideration, Gonorrheal Infection in Women, has come to be one of the first importance in view of the light—clinical, pathological, and histological—thrown upon it by the labors of Noeggerath and Neisser. It is doubtful if any disease is capable of propagating more ot sickness, woe, and misery as an uncured gonorrhea. In view of the recent researches, and especially of the clinical experiences of abdominal surgeons, it seems to be admitted that what was taught and written on the treatment of this infection prior to 1872, was little better than so much chaff. Nor did the medical profession heed the notes of warning sounded by Noeggerath when he published his "opuscule" in 1872. It has taken fifteen years of "continual hammering" to arouse the profession to the dangers that lurk in an uncured or latent gonorrhea. Most surgeons or physicians who have been asked to treat the disease in its acute stages, whether in the male or female, have generally regarded it in a light or trifling manner, contenting themselves with ministering to the first distress, and paying little heed to its secondary possibilities. The literature of this disease must be entirely rewritten, and the teachings of the schools entirely recast, in view of the awful consequences of neglect, to entirely stamp out or prevent the propagation of this subtle, deceptive and virulent infection, when once it has been contracted.

It has come to a pass that a woman who accepts an offer of marriage finds herself treading upon dangerous ground—a danger far more extended than by any possible contemplation of mother Eve when she entailed her sex—and it becomes a problem of the most serious import, when her answer may fetch her to "a spring of woes unnumbered." We have been impelled to say this much by a sense of duty that has been growing for some time upon us, and the monograph before us serves to again admonish us that the fulness of time has arrived when some serious talk must be had on the subject.

In the introductory chapter of the brochure, Dr. Sinclair strongly brings out the importance of a clear knowledge of his subject, not only to the gynecologists whom he mainly aims to reach, but to every practiser of the medical art. He shows the inadequacy of the teaching of text-books on the subject in a most striking manner. In the historical retrospect, in Chapter II., our author divides his subject chronologically into three epochs, namely: First, before Noeggerath's treatise appeared; second, Noeggerath's work and its immediate influence; and third, Neisser's discovery. In the first period is included all the centuries from Hippocrates down to the threshold of the last quarter of the nineteenth century. The second period covers but seven years, namely, from 1872 to 1879; and the third period embraces the years subsequent to Neisser's discovery of the gonococcus.

The discovery of this micro-organism furnishes a pivot around which diagnosis may swing with certainty. For we are not aware that the accuracy of Neisser's discovery has been successfully challenged by any competent authority.

Chapter III., devoted to Contemporary Pathology and the "Gonococcus-Neisser," is a most interesting one, and deserves to be thoroughly studied in connection with pathological researches of the malady.

In Chapter IV., devoted to Clinical Phenomena, the interest is heightened by the cases cited in detail in support of the position assumed.

Chapter V. is devoted to the Consequences of Gonorrheal Infection, and we wish it could be furnished to every young man of proper age in the land. We mean, of course, its essential features, divested of its technical and scientific phraseology.

The final chapter (VI.) considers Treatment and Prophylaxis, which concludes a most interesting, instructive, and classical treatise of 128 pages, on this important subject. We have nothing but praise to give our author who, jointly with the publishers, is entitled to great commendation for placing this valuable monograph within the range of every physician's purse.

The remaining subjects considered in this number we reserve for a future notice.

